# SARS-CoV-2 mRNA Vaccine Effectiveness in the Borriana COVID-19 Cohort: A Prospective Population-Based Cohort Study

**DOI:** 10.3390/epidemiologia7010001

**Published:** 2025-12-19

**Authors:** Salvador Domènech-Montoliu, Óscar Pérez-Olaso, Diego Sala-Trull, Alba Del Rio-Gonzalez, Laura López-Diago, Isabel Aleixandre-Gorriz, Maria Rosario Pac-Sa, Manuel Sánchez-Urbano, Paloma Satorres-Martinez, Cristina Notari-Rodriguez, Juan Casanova-Suárez, Raquel Ruiz-Puig, Gema Badenes-Marques, Laura Aparisi-Esteve, Carmen Domènech-León, Maria Angeles Romeu-Garcia, Alberto Arnedo-Pena

**Affiliations:** 1 Medical Direction Department, University Hospital de la Plana, 12540 Vila-Real, Spain; domenech_salmon@gva.es; 2 Microbiology Service Department, University Hospital Doctor Peset, 46027 Valencia, Spain; perez_oscola@gva.es; 3 Emergency Service Department, University Hospital de la Plana, 12540 Vila-Real, Spain; saladiego2@gmail.com (D.S.-T.); manu.msu@gmail.com (M.S.-U.); palomasatmar@gmail.com (P.S.-M.); notari_cri@gva.es (C.N.-R.); raquelruizpuig@gmail.com (R.R.-P.); gemabamar@hotmail.com (G.B.-M.); 4 Health Center I, 12530 Borriana, Spain; delrio_alb@gva.es; 5 Clinical Analysis Service Department, University Hospital de la Plana, 12540 Vila-Real, Spain; lopez_laudia@gva.es (L.L.-D.); aleixandre_isagor@gva.es (I.A.-G.); 6 Public Health Center, 12003 Castelló de la Plana, Spain; charopac@gmail.com (M.R.P.-S.); aromeu96@gmail.com (M.A.R.-G.); 7 Nursing Service Department, University Hospital de la Plana, 12540 Vila-Real, Spain; juancasanova83@gmail.com; 8 Carinyena Health Center, 12540 Vila-Real, Spain; lauraaparisiesteve@gmail.com; 9 Department of Medicine, Universidad CEU Cardenal Herrera, 12006 Castelló de la Plana, Spain; carmendomenech04@gmail.com; 10 Epidemiology and Public Health (CIBERESP), 28029 Madrid, Spain; 11 Department of Health Science, Public University Navarra, 31006 Pamplona, Spain

**Keywords:** COVID-19, SARS-CoV-2 infection, SARS-CoV-2 mRNA vaccines, vaccine effectiveness, symptomatic, asymptomatic, prospective cohort, population-based

## Abstract

Background and Objective: Evaluating vaccine effectiveness (VE) is essential to implementing prevention strategies, and our objective was to estimate the VE of SARS-CoV-2 messenger RNA (mRNA) vaccines in preventing SARS-CoV-2 infection. Materials and Methods: We carried out a population-based, prospective cohort study on the Borriana COVID-19 cohort (Valencia Community, Spain) during the 2021–2023 period, considering all SARS-CoV-2 cases that occurred after the SARS-CoV-2 vaccine campaign started in January 2021 (first approach), as well as only symptomatic cases (second approach). Multivariable robust Poisson regression models were employed. Results: In this cohort with 301 participants, 285 were vaccinated, among whom 228 received only SARS-CoV-2 mRNA vaccines, and 57 received mRNA vaccines and other vaccines. In the first approach, there were 226 cases and 75 non-cases. The adjusted VE for three doses of vaccine was 37% (95% confidence interval [CI]: 22–49%) to prevent infection. In the second approach, with 153 symptomatic cases after excluding 73 asymptomatic cases, the adjusted VE for three doses of vaccine was 50% (95% CI 33–63%) to prevent symptomatic infection. Three doses of vaccine exhibited modest but significant protection against infection and symptomatic infection. Conclusions: This study recommends surveilling SARS-CoV-2 infections and variants, vaccinating at-risk populations, and developing new vaccines.

## 1. Introduction

SARS-CoV-2 vaccines have been evaluated extensively for their effects on COVID-19 severity, hospitalizations, deaths, and long COVID incidence, highlighting their protective results [1,2,3,4,5]. However, SARS-CoV-2 infection has not been evaluated as extensively, considering the new virus variants and the waning immunity [6,7].

Quantifying SARS-CoV-2 vaccine effectiveness (VE) has been considered essential from a public health perspective to tackle the pandemic situation [8]. However, an estimation of VE presents some difficulties, requiring considering the demographic, clinical, and socio-economic context, follow-up of the vaccinated population, measuring SARS-CoV-2 exposures, and taking into account the high proportion of asymptomatic infections, the prevalence of infected populations before vaccination, SARS-CoV-2 variants, and the different SARS-CoV-2 vaccines and doses received. This complex situation could lead to bias in the VE estimation due to selection and confounding biases, among other reasons [9]. In addition, different effectiveness estimation methods have been employed [10].

The objective of this study was to estimate the VE of SARS-CoV-2 messenger RNA (mRNA) vaccines to prevent SARS-CoV-2 infections in the Borriana COVID-19 cohort during the period from January 2021 to August 2023.

## 2. Materials and Methods

### 2.1. Study Design

We carried out a prospective population-based cohort study of individuals who were infected during the Borriana COVID-19 Fallas festival in March 2020 in Borriana, a city in the Castellon province within the Valencia Community (Spain), where a large outbreak of COVID-19 took place, with an attack rate of 46% [11]. Several studies on this cohort with follow-up have been published from 2021 to 2025, considering the persistence of SARS-CoV-2 antibodies, complications, risk factors, long COVID, and cellular immunity [12,13].

In June 2022, a third study was performed on this cohort, including participants with laboratory-confirmed COVID-19; 722 of 1132 participants (63.8%) were included in the study. This study conducted serologic and questionnaire surveys to determine the SARS-CoV-2 infection and vaccination status. In addition, a follow-up of the cohort was implemented from June 2020 to August 2023 by examining their healthcare records. The present research covered the period from January 2021, when the SARS-CoV-2 vaccination campaign, to August 2023, when a survey of the primary healthcare record of each participant was conducted. 

The inclusion criteria for this study were that participants had a negative laboratory-confirmed test of COVID-19 in previous surveys of this cohort (11–12) and that they did not suffer from the SARS-CoV-2 infection before the start of the SARS-CoV-2 vaccination campaign in January 2021. Asymptomatic SARS-CoV-2 cases were included if laboratory confirmation tests were performed after the start of the vaccination campaign in January 2021. Only participants with a first SARS-CoV-2 infection were considered, and reinfections were studied. Participants who suffered COVID-19 infection before the vaccination campaign were excluded, based on a follow-up of the cohort from 2020 onwards [11,12]. In addition, participants without laboratory-confirmed infection were excluded.

**Figure 1:**
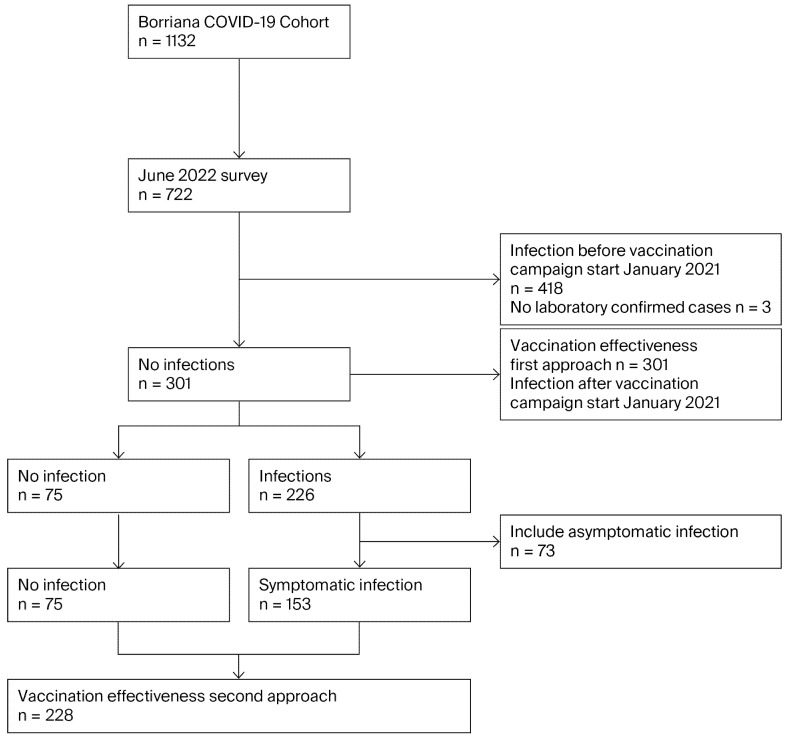
Flow diagram of SARS-CoV-2 infections and vaccine effectiveness in Borriana COVID-19 cohort.

Two approaches were performed to estimate the VE of SARS-CoV-2 mRNA vaccines among the participants included in this study: all SARS-CoV-2 infections, including both symptomatic and asymptomatic cases (first approach), and only SARS-CoV-2 symptomatic infections (second approach). Symptomatic infections were defined by the presence of at least one illness symptom, including fever, coryza, cough, headache, myalgia, sore throat, weakness, loss of smell and/or taste, dyspnea, diarrhea, vomiting, and laboratory-confirmed tests. Asymptomatic infections were defined as the absence of any symptoms of SARS-CoV-2 infections, confirmed through laboratory tests. No SARS-CoV-2 infections were defined as the absence of any symptoms of SARS-CoV-2 infections and negative laboratory SARS-CoV-2 tests. SARS-CoV-2 cases were participants who suffered asymptomatic or symptomatic SARS-CoV-2 infections, and non-cases were participants who did not suffer SARS-CoV-2 infections. Figure 1 shows the flow diagram of this study.

To estimate VE, it is important to distinguish between asymptomatic and symptomatic cases, since it is unknown when asymptomatic cases suffered the infection, and there is a high proportion of asymptomatic cases from SARS-CoV-2 infections [14]. Therefore, whether the vaccination was before or after the infection cannot be determined. The first approach included all SARS-CoV-2 infections, and the second approach estimated VE based on symptomatic infections only. The use of these two approaches could estimate VE with higher precision by considering how viral transmission was passed over a more extended period of the pandemic, allowing us to gain better insight into the pandemic [15]. 

Considering the initial date of the vaccination campaign in January 2021 and the final date of the study in August 2023, the follow-up time of this cohort was 36 months. In this period, the predominant SARS-CoV-2 variants in the study area were Alpha from January 2021 to July 2021, Delta from August 2021 to December 2021, and finally Omicron from January 2021 to August 2023 [16].

### 2.2. SARS-CoV-2 Vaccination

The onset of the SARS-CoV-2 vaccination began in January 2021, and the last vaccinations took place in February 2022. Then, a participant was considered vaccinated 14 days after receiving one, two, or three vaccine doses [17]. To estimate the number of unvaccinated participants, medical and vaccination records were consulted.

Regarding the type of SARS-CoV-2 vaccines, most participants in the cohort received (228 participants) mRNA vaccines, including Pfizer-BioNTech (BNT162b2) (Pfizer, New York, NY, USA) (Comirnarty^®^) for the first and second doses, and Moderna (mRNA-1273) (Moderna, Cambrige, MA, USA) (Spikevax^®^) for the third dose. In addition, the AstraZeneca (AZD1222) (AstraZeneca, Cambridge, UK) (Vaxzevria^®^ Covishiel^®^) vaccine was administered to 54 participants for the first and second doses. A first dose of the Janssen^®^ SARS-CoV-2 vaccine (Ad26.CoV2.S) (Johnson and Johnson Innovative Medicine, Titusville, NJ, USA) (Jcovden^®^) was administered only to 3 participants. A third dose of the SARS-CoV-2 mRNA vaccine was administered to all participants. Information about vaccine brand and vaccination dates was obtained by consulting the population-based Valencia Region Vaccine Information System.

### 2.3. Laboratory Confirmation Tests for SARS-CoV-2 Infections

Laboratory confirmation tests for SARS-CoV-2 infections for the cohort began in May 2020 [11] with the determination of antibodies against SARS-CoV-2 nucleocapsid protein N via electrochemiluminescence immunoassay [18] (Roche Diagnostics, Mannheim, Germany). In the serologic survey conducted in June 2022, anti–SARS-CoV-2 spike IgG antibodies and IgG and IgM anti-nucleocapsid antibodies were detected via chemiluminescence microparticle immunoassay (CMIA AlinityI series, Abbot, Chicago, IL, USA) [19]. During the follow-up of this cohort, for the detection of the SARS-CoV-2 virus, reverse transcription–polymerase chain reaction (RT-PCR) was employed on different platforms from various brands: Genexpert, Roche Diagnostics, and Simplexa (Barcelona, Spain), as well as rapid antigen tests (RATs). Serology tests and the RT-PCR were performed by the Laboratory of Microbiology Service of the University Hospital de la Plana (Vila-real, Spain), and RATs by the participants at home.

The laboratory tests presented the following sensitivity and specificity values: CMIA, 96–98% sensitivity and 100% specificity [18]; RT-PCR, 99–100% sensitivity and 99–100% specificity [20]; and RATs (according to different brands frequently used in Spain), 80.2–96% sensitivity and 98.9–99.6% specificity [21]. SARS-CoV-2 infection status was determined for positive cases using the following laboratory tests: CMIA, 136 cases (60.2%); RT-PCR, 17 cases (7.5%); and RATs, 73 cases (32.3%). The status for non-cases was determined using CMIA, confirming 75 cases (100%). 

### 2.4. Research Surveys

Three surveys were performed from June 2020 to June 2022 [22]. Information was obtained through specific questionnaires about socio-demographic characteristics; cohabitants at home; chronic disease; body mass index (BMI) (kg/m^2^); lifestyle, including smoking habits, alcohol consumption, and physical exercise; history of SARS-CoV-2 infections, including date of onset; and risk factors for SARS-CoV-2 exposure, including family member COVID-19 cases, exposure to other people at work, restaurant/bar attendance, and face mask wearing. Face-to-face interviews and telephone surveys were conducted by health staff from the Primary Care Health Center of Borriana and Vila-real, the Emergency Service of University Hospital de la Plana, and the Public Health Center of Castellon.

In addition, the primary healthcare record of each participant was reviewed and revised accordingly, and the computerized application outpatient care of the Valencia Health Agency (ABUCASIS) was queried from January 2020 to August 2023 to detect new COVID-19 cases, reinfections, and disease evolution.

### 2.5. Statistical Methods

Statistical methods for the two approaches were similar. Means, standard deviations, and percentages were used to describe the characteristics of the studied population. The cumulative incidence rate was estimated by dividing the SARS-CoV-2 cases by the total participants, and the 95% confidence interval (CI) was estimated according to a binomial distribution. Relative risk (RR) was defined as the ratio between the incidence rate of SARS-CoV-2 infection in exposed participants and the incidence rate of SARS-CoV-2 in unexposed participants, considering the different variables. Crude and adjusted relative risk (aRR) were estimated, with 95% CI.

SARS-CoV-2 infection and SARS-CoV-2 mRNA vaccination were the dependent and predictor variables, respectively, with several independent variables adjusted in the analysis using robust Poisson regression models [23]. These models exhibited robust standard errors that could correct for the high variability in the data. All models fitted well based on the Pearson and chi2 tests. Computer outputs are found in Supplementary S1.

**Figure 2:**
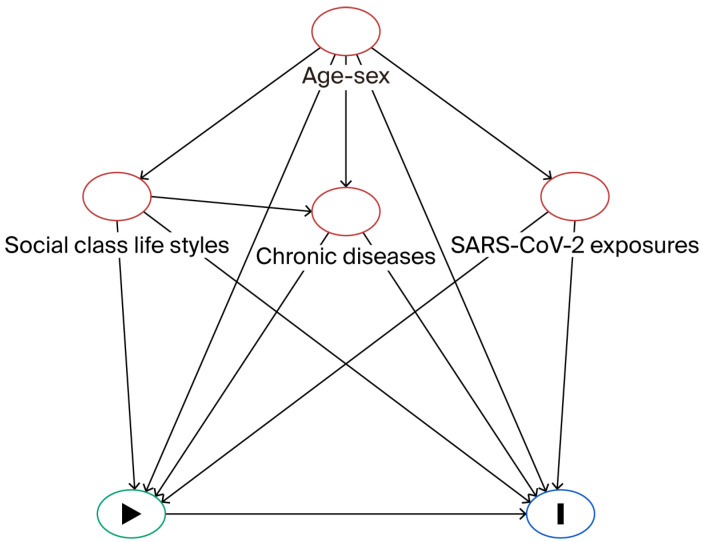
Directed Acyclic Graph (DAG) of SARS-CoV-2 vaccine (exposure) and SARS-CoV-2 case (outcome). Ancestor of exposure and outcome (in red) based on the DAGitty version 3.1 program. Potential confounding factors: age, sex, cohabitants at home, social class, chronic diseases, lifestyles (smoking, obesity, alcohol consumption, physical exercise), and SARS-CoV-2 exposures (family member with COVID-19, exposure to other people at work, visiting restaurants/bars, and face mask wearing).

Vaccine effectiveness (VE) was calculated as (1-RR) × 100 [24] from the aRR. To control for potential confounding factors, Directed Acyclic Graph (DAG) methodology was conducted [25,26] using the DAGitty version 3.1 program [27] (Figure 2). Confounding factors included age, sex, cohabitants at home, social class (considering upper and middle class (I–II) versus lower class (III–VI)), lifestyles (smoking, obesity as BMI ≥ 30 kg/m^2^, alcohol consumption, physical exercise), chronic diseases, and SARS-CoV-2 exposures (family member with COVID-19, exposure to other people at work, visiting restaurants/bars, face mask wearing) [28]. A crude RR of each of these factors in relation to SARS-CoV-2 infection was determined. In addition, adjusted VE was estimated for stratification, considering sex, age 50 years and above, and the presence of chronic diseases. The Stata^®^ version 14 program(StataCorp., College Station, TX, USA) was used for all calculations.

### 2.6. Ethical Issue

This study was submitted to and approved by the Ethics Committee of University Hospital de la Plana (14 October 2021; registry number: 2961). All participants, or their parents in the case of minors, provided informed written consent to be included in the study.

## 3. Results

### 3.1. Follow-Up of the Borriana COVID-19 Cohort from March 2020 to August 2023

Participants of the initial Borriana COVID-19 cohort with laboratory-confirmed COVID-19 (*n* = 1132) were invited to participate in the June 2022 survey, and 722 were enrolled, yielding a participation rate of 63.8% (722/1132). From March 2020 to August 2023, 644 participants were infected with SARS-CoV-2, as confirmed by a laboratory test. Three participants had suspected infection but without laboratory confirmation, and 75 participants did not suffer an infection, yielding a cumulative incidence rate of 89.2% (95% CI: 86.7–91.4%) (644/722).

Participants who suffered a SARS-CoV-2 infection before the vaccination campaign in January 2021 and participants without a laboratory-confirmed test were excluded. From January 2021 to August 2023, out of 301 participants, 226 participants suffered a SARS-CoV-2 infection and 75 participants did not. Among the cases, 73 were asymptomatic and 153 were symptomatic. The cumulative incidence rate was 75.1% (95% CI: 69.8–79.9%) (226/301).

Clinical characteristics of the symptomatic cases included fever (59.4%), coryza (46.2%), cough (45.8%), headache (43.4%), myalgia (38.5%), sore throat (34.3%), weakness (33.6%), loss of smell and/or taste (14.2%), dyspnea (11.9%), diarrhea (9.2%), and vomiting (6.3%). The average disease duration was 5.7 ± 4.2 days, with a median of 5.0 days (rank 1–30). Long COVID sequelae were reported in 28 cases (12.4%). A total of 102 cases sought medical assistance (66.7%), and 6 cases were hospitalized due to COVID-19 (3.9%). In the study period, one 72-year-old woman died two months after the onset of SARS-CoV-2 infection, but the cause was not associated with the infection.

**Figure 3:**
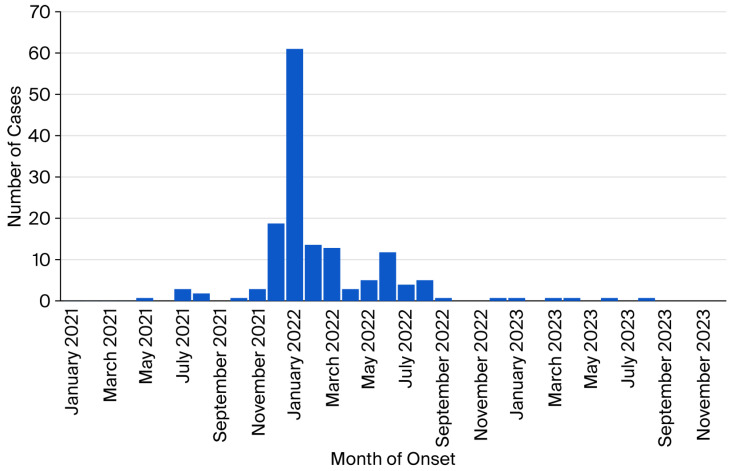
Temporal distribution of SARS-CoV-2 cases by symptom onset.

The temporal distribution of the 153 cases by symptom onset is shown in Figure 3. The onset of the first COVID-19 case occurred in May 2021, with an increase in cases from July to December 2021 (18.2%), a peak of cases in January 2022 (39.6%), followed by a slow decline up to August 2022 (37.7%), and the last case occurred in August 2023. Most cases were probably caused by Omicron variants.

Crude RRs were estimated to compare SARS-CoV-2 cases and non-cases (Table 1). Cases were significantly younger than non-cases, with the proportion of subjects aged 50 and over being lower. Males presented a lower risk compared with females (RR = 0.87; 0.95% CI: 0.75–0.84). Social class, obesity, and chronic disease were not risk factors for SARS-CoV-2 incidence. Alcohol consumption and physical exercise also had no effect on SARS-CoV-2 incidence. In contrast, smoking status had a protective effect (RR = 0.76; 95% CI: 0.60–0.97). A high number of cohabitants at home (RR = 1.08; 95% CI: 1.02–1.16) and exposure to other people at work (RR = 1.20; 95% CI: 1.00–1.43) were risk factors for infection. Even though having a family member with COVID-19 and visiting restaurants and bars presented an increased risk of SARS-CoV-2 infection, their effect was not significant. Face mask wearing was a protective factor, but it was not significant.

### 3.2. The First Approach Included Participants Vaccinated Before Suffering from a SARS-CoV-2 Infection

This study included 301 participants, with 226 SARS-CoV-2 cases and 75 non-cases. Among the cases, 73 were asymptomatic and 153 were symptomatic. In the Borriana COVID-19 cohort, SARS-CoV-2 vaccination began in January 2021, and 57.9% (418/722) of the cohort had already suffered from COVID-19 disease. Table 2 presents the results of SARS-CoV-2 vaccination in cases and non-cases. Of the 301 participants, 285 (94.7%) were vaccinated against COVID-19 disease with at least one dose, 275 (91.4%) with two or three doses, and 165 (54.8%) with three doses. Only 16 participants were not vaccinated. SARS-CoV-2 mRNA vaccines were exclusively administered to 228 participants (80%), and 57 participants (19%) received mRNA vaccines and other SARS-CoV-2 vaccines (AstraZeneca and Janssen). This highlights the high proportion of vaccinated individuals (92.9%) among SARS-CoV-2 cases. However, vaccination was a significant protective factor against SARS-CoV-2 infection, specifically with at least one dose (RR = 0.74 95% CI 0.69–0.79), with a complete vaccination (two to three doses) (RR = 0.72; 95% CI: 0.68–0.78), and with a booster vaccination (three doses) (RR = 0.68; 95% CI: 0.59–0.77). Comparing the number of vaccine doses administered, two and three doses of vaccines were protective against SARS-CoV-2 infection, with (RR = 0.89; 95% CI: 0.83–0.95) and (RR = 0.62; 95% CI: 0.55–0.70), respectively. No significant differences were found when comparing types of vaccines.

The adjusted relative risks (aRR) of SARS-CoV-2 vaccine doses and VE are presented in Table 3. The ARRs showed that vaccination was significantly protective against SARS-CoV-2 infections, considering vaccinated versus unvaccinated (aRR = 0.78; 95% CI: 0.63–0.96), vaccinated 2–3 doses versus 0–1 dose (aRR = 0.82; 95% CI: 0.70–0.95), and vaccinated with 3 doses versus 0–1–2 doses (aRR = 0.71; 95% CI: 0.61–0.82). Considering the number of vaccine doses, three doses were significantly protective (RR = 0.63; 95% CI: 0.61–0.82), whereas two doses were not significantly protective. The VE was 22% in vaccinated versus unvaccinated individuals (95% CI: 4–37); 18% in individuals vaccinated with 2–3 doses (95% CI: 5–30); and29% in individuals vaccinated with 3 doses (95% CI: 18–39). Regarding the number of vaccine doses, three doses had a VE of 37% (95% CI: 22–49), and two doses had a VE of 11% (−3–24).

The results of SARS-CoV-2 vaccine effectiveness stratified by sex, chronic disease, and age of 50 years and over are presented in Table 4. Males had a lower aRR for suffering from a SARS-CoV-2 infection than women (0.62 versus 0.74), and their VE was higher, at 38% versus 26%. Participants with a chronic disease had a lower aRR than participants without a chronic disease (0.60 versus 0.74), and their VE was higher, at 40% versus 26%. Participants aged 50 years and over had a lower aRR than participants aged less than 50 years (0.51 versus 0.77) and a higher VE of 49% versus 23%.

### 3.3. The Second Approach Excluded Participants with Asymptomatic SARS-CoV-2 Infections

Among the 226 SARS-CoV-2 cases in the first approach, 153 participants suffered from a symptomatic SARS-CoV-2 infection. In 73 participants, the infections were asymptomatic, and thus, these participants were excluded from this analysis. Asymptomatic cases represented 32.3% of cases (73/226), and among them, 66 participants were vaccinated (90.4%). In the second approach, there were 228 participants, including 153 cases and 75 non-cases.

The non-cases were significantly older than the cases, and males had a lower risk of SARS-CoV-2 infection (Table 5). Social class, chronic disease, alcohol intake, obesity, and physical exercise were not risk factors for infection. However, current smoking was a significant protective factor (RR = 0.76; 95% CI: 0.60–0.97). The number of cohabitants at home (RR = 1.14; 95% CI: 1.04–1.25) was a risk factor, as well as exposure to other people at work (RR = 1.30; 95% CI: 1.01–1.68). Having a family member with COVID-19 and visiting restaurants/bars increased the risk of infection, though their effect was not significant, and face mask wearing was not a significant protective factor.

Of the 228 participants, 219 were vaccinated, and 153 were cases while 75 were non-cases (Table 6). Only nine cases were unvaccinated. Vaccination was significantly protective against SARS-CoV-2 symptomatic infections, specifically with at least one dose (RR = 0.66; 95% CI: 0.60–0.72), with two or three doses (RR = 0.64; 95% CI: 0.58–0.71), and with three doses (RR = 0.51; 95% CI: 0.42–0.64). Considering the number of doses, three doses yielded higher protection (RR = 0.46; 95% CI: 0.38–0.56) than two doses (RR = 0.87; 95% CI: 0.81–0.94). No differences were observed between receiving an mRNA vaccine alone versus receiving mRNA and other vaccines.

The adjusted RR and VE of SARS-CoV-2 vaccines for the symptomatic cases are presented in Table 7. Receiving one dose of SARS-CoV-2 vaccine did not protect against symptomatic SARS-CoV-2 infection (aRR = 0.81; 95% CI: 0.53–1.22); the same was observed for vaccination schedules with two and three doses (aRR = 0.82; 95% CI: 0.67–1.01), but three-dose vaccination was a significant protective factor (aRR = 0.54; 95% CI: 0.44–0.68), with a VE of 46% (95% CI: 32–56). Considering the number of doses, three doses were significantly protective against symptomatic SARS-CoV-2 infection (aRR = 0.50; 95% CI: 0.37–0.67), with a VE of 50% (95% CI: 33–63). Two doses of vaccine were not significantly protective against symptomatic SARS-CoV-2 infection.

The results of SARS-CoV-2 vaccine effectiveness for symptomatic cases stratified by sex, chronic disease, and age of 50 years and over are presented in Table 8. Males had a lower aRR than women (0.38 versus 0.61) and a higher VE (62% versus 39%). Participants with a chronic disease had a lower aRR than participants without a chronic disease (0.48 versus 0.55) with a higher VE (52% versus 45%). Participants aged 50 years and over had a lower aRR than participants under 50 years old (0.40 versus 0.61), and their VE was higher (60% versus 39%).

### 3.4. Comparisons of aRR and VE Between the First and Second Approaches

Comparisons of aRR and VE between the first and second approaches are presented in Table 9. In both approaches, vaccination with three doses significantly reduced the risk of SARS-CoV-2 infections, with an aRR of 0.63 (first approach) and 0.50 (second approach), and the VE was 0.37%, and 0.50%, respectively. In the two approaches, vaccination with only one or two doses was not protective. After stratification, the second approach reduced the aRR compared with the first approach, especially in male participants and participants aged 50 years and over. The VE increased with the second approach.

## 4. Discussion

Three years after the beginning of the COVID-19 pandemic in 2020, 89.2% of the cohort suffered a SARS-CoV-2 infection, and 75.1% after the vaccination campaign started in January 2021, showing the magnitude of the pandemic. Despite a high vaccination rate with mRNA vaccines in most participants, with 94.6% receiving at least one dose and 54.8% receiving three doses, the incidence of SARS-CoV-2 infection was high. However, in both approaches, vaccination gave significant protection against SARS-CoV-2 infection when three doses were administered. In the first and second approaches, the VE was 37% for infection and 50% for symptomatic infection, respectively. These two VE values are modest, but in line with the increase in VE in cases with severe illness, hospitalizations, and deaths reported in cohort studies; however, in the Omicron wave, severe outcomes decreased [29,30,31,32]. Moreover, a reduction in the VE of mRNA vaccines related to the number of doses during the Omicron wave was found in previous cohort and meta-analysis studies [33,34]; three doses significantly decreased the risk of infection and symptomatic infection, but two doses were not sufficient to reduce the risk.

The VE values estimated by the two approaches may reflect the spectrum in which VE moves. The VE of the first approach should be close to the true value of the VE that occurred in the community since it included all cases with an infection. In the second approach, the VE included cases with symptomatic infections only. In this regard, Williams and coauthors [35] mentioned that the inclusion of asymptomatic cases reduces the VE in vaccines that prevent disease but not infection. In test-negative design studies of VE, the inclusion of asymptomatic cases could bias the VE [36].

Regarding three vaccine doses, results of the two approaches with stratification highlight a higher VE in males, participants with chronic diseases, and participants 50 years old and above. This could correspond with a higher incidence of Omicron variant infections in young people [37]. As a consequence, the VE is higher in older participants and participants with chronic conditions, although the vaccine response decreases with age and chronic conditions [38]. A higher VE in males versus females has also been found in other cohort studies, but the reasons are not well understood [39], since the immune response is lower in males [40].

To understand our results, it is important to consider the long duration of the follow-up and the SARS-CoV-2 variants during this period, with the Omicron variant being predominant for most of the study period. Two variables, time from vaccination and virus variants, are crucial for SARS-CoV-2 risk infection quantification and VE. In initial clinical trials of SARS-CoV-2, the VE during the first 6 months was very high, reaching 96%, and declined progressively afterwards. The protection against infection declined 20–30 percentage points [2,41] 6 months after vaccination. In addition, new variants of SARS-CoV-2 led to an increase in breakthrough infections in vaccinated populations [42,43]. Other factors could also contribute, including a decrease in non-pharmacological measures against infection 2 years after the onset the COVID-19 pandemic and vaccine reluctance [44]. Very high SARS-CoV-2 vaccination rates might be necessary to stop SARS-CoV-2 transmission, considering reproductive numbers between 5.8 and 6.1 [45]. In addition, vaccine breakthrough infections allowed for continued transmission, given that vaccinated cases had viral loads similar to unvaccinated cases [46], although infection duration could be shorter in vaccinated subjects [47].

In this context, comparing our results with those of other cohort studies reveals that the effectiveness of mRNA vaccines in preventing SARS-CoV-2 infection after the first epidemic period from 2020 to 2021 decreased against the Omicron variant. Regarding symptomatic SARS-CoV-2 cases, the VE was 47.9% (95% CI: 41.85–53.75) in a review study [48]. Among healthcare workers in a clinic in Cleveland, the decline in VE was from 49% to 19% across the periods considered [49]. In Spain, Monge and co-authors found VE values of 52·5% (95% CI: 51.3–53.7%) for an mRNA-1273 booster and 46.2% (95% CI: 43.5–48.7%) for a BNT162b2 booster in a national cohort [50]. In Hong Kong, the BNT162b2 vaccine was protective against asymptomatic and symptomatic SARS-CoV-2 Omicron infections with a VE of 41.4% (95% CI 23.2–55.2%); the VE of BNT162b2 boosters was 50.9% (95% CI 31%·65%) for symptomatic Omicron infections [51]. In Japan, a cohort study on a general population of 16- to 64-year-olds found that the mRNA vaccine had a VE of 71.8% (95% CI: 60.1–80.1%) for a third dose against symptomatic infection during the Omicron period [52]. In Canada, a cohort study on healthcare workers found a VE of 43% (95% CI: 29–54%) for three doses, and 56% (95% CI: 42–67%) for four doses, after adjusting for previous infection and other covariates [53].

In our study, we controlled for potential risk factors, including demographics, lifestyles, virus exposure, and prevention measures, as recommended for VE studies [54,55]. However, some authors reported that exposure had little effect on VE [17]. Regarding related factors of SARS-CoV-2 infections, our study found that older and male participants had a lower risk of infection. On the contrary, the number of cohabitants at home and exposure to other people at work increased the risk of infection, while current smoking was a protective factor. Other lifestyle factors and preventive measures against virus transmission had no effect on the risk of infection. Some of these factors have been reported in studies on SARS-CoV-2 epidemiology, such as a higher risk of infection for female sex and larger household size [56], young age [57], exposure to other people at work as a proxy for working outside the home [58], and certain education and social care occupations [59]. Current smoking was a protective factor, in line with other studies [60]. Wearing a face mask was not protective, which is in line with a prospective cohort study in Germany [61], but this factor was found to be protective in other studies [62]. Moreover, obesity was not a risk factor, in contrast to what was found in England [63].

To explain the low VE in preventing SARS-CoV-2 infection, some studies have indicated that the intramuscular injection of mRNA vaccines could be the cause of low IgG antibody levels in the upper respiratory tract, in contrast with higher antibody concentrations in the circulation [64]; other factors may be the high frequency of SARS-CoV-2 variants, including the Omicron variant with a predilection for the upper respiratory tract, and the rapid decrease in neutralizing antibodies against this variant with a need for vaccine boosters to increase these antibodies [65,66]. Regarding the reduction in severe events, its biological mechanism is not completely understood. In addition to neutralizing antibodies with immune memory T cells and B cells to control the disease, a hypothesis has been proposed that mRNA vaccines reduce the activation of alveolar macrophages, decreasing the release of cytokines, with some side effects [67].

The study of VE is critical for the implementation of strategies to control and prevent COVID-19 epidemics, but it is subject to a high likelihood of bias, considering the design, performer, and results. Several reviews have mentioned these issues, including case–control, test-negative, and cohort studies [68,69,70]. In addition, the estimation of the VE duration still requires accurate analysis [36]. Our study has strengths and limitations. Regarding strengths, this study employed a prospective, population-based cohort design with long follow-up, considering laboratory-confirmed cases; it used an official vaccine register, controlled for potential confounding factors, and employed two approaches to measure the VE of SARS-CoV-2 vaccines, one including all incident cases and one including only symptomatic cases. Regarding the laboratory tests used to determine the SARS-CoV-2 infection status, CMIA was used in 70.1% of participants, RT-PCR in 5.6%, and RATs in 24.3, with the last test showing inferior sensitivity. However, the sensitivity and specificity of all tests conformed to the recommendations of the World Health Organization, such as sensitivity ≥80 and specificity ≥97–100% [71]. With respect to the adjusted VE of three doses for the two approaches, we calculated the E-values [72] to be 2.55 (upper limit of 1.89) and 3.41 (upper limit of 2.35) for the first and second approach, respectively, indicating a low magnitude of unmeasured confounding, which is in line with other studies on VE [73].

This study’s limitations are as follows: First, the number of participants in the cohort is small. Second, SARS-CoV-2 variants were not detected. Third, exposure measures were determined through a questionnaire retrospectively in some infected cases. Fourth, non-cases might have suffered a SARS-CoV-2 infection after the June 2022 survey, but the healthcare records of each participant were revised up to August 2023, and the survey of a random sample of 225 participants to study the cellular immunity of SARS-CoV-2 [13] could reduce this quantity. Fifth, there was a reduction in statistical power in the second approach. Sixth, the analysis of potential risk factors was not adjusted. Seventh, a portion of participants received two types of vaccines against SARS-CoV-2. Eighth, some other residual confounding factors could remain. Ninth, the decline in IgG and IgM anti-nucleocapsid antibodies over time in SARS-CoV-2 cases could increase the VE, since some asymptomatic cases could be non-cases. Tenth, given their lower sensitivity, RATs might not have detected all infected cases. Eleventh, COVID-19 is a new disease, and some issues might not have been considered in this study. Finally, the design of our study is observational and has inherent limitations compared to clinical trial designs [9].

Regarding the interpretation of our results, which show modest but significant protection against SARS-CoV-2 infection after three doses of vaccination, following Austin Bradford Hill’s causality criteria [74], a causal relationship, rather than association, could be suggested between mRNA vaccine and SARS-CoV-2 infection, considering the prospective design, consistency with other studies, biological plausibility, coherency, dose–response, and specific immune response; in addition, confounding factors were controlled. However, it is necessary to account for the fact that our study was observational with possibility of bias. 

The administration of mRNA vaccines against SARS-CoV-2 infection contributed to a substantial reduction in the impact of the pandemic, decreasing the incidence of severe events, but viral transmission could not be completely prevented. Our VE results should be considered in a context where reduced risk of disease via vaccination is evident, but vaccine failure is present and viral transmission continues. An outbreak ends when a high proportion of exposed people are infected, and this situation needs to be considered in vaccination campaigns [75]. In addition, there are controversies regarding the effectiveness of vaccination campaigns and the safety of vaccines [76,77,78]. In this regard, the follow-up of this cohort could provide useful information regarding the evolution of the disease, vaccinations, and side effects.

To address the activity and high-frequency mutation of the virus with a decrease in VE, several measures need to be considered, including surveillance of the incidence of SARS-CoV-2 infections and virus variants, research on the biology of the virus and immune response of vaccines, and vaccination of the at-risk population [79,80]. The WHO recommends a single-dose regimen for primary vaccination against COVID-19, suggesting that the monovalent Omicron XBB vaccines could provide better protection than other vaccines, and revaccination for groups with severe disease and death [81,82]. In Europe, people over 60 years of age, people with immunodeficiency, and pregnant women should be vaccinated but with different administration schedules [83].

In addition, the development of new vaccines against SARS-CoV-2, including a pan-coronavirus vaccine, with long-term protection is a priority for public health strategies to prevent new COVID-19 epidemics [84,85].

## 5. Conclusions

The results of this study suggest that SARS-CoV-2 mRNA vaccines have modest protection against SARS-CoV-2 infections and symptomatic infections after three doses. One or two doses are insufficient to protect against infection. Thus, we recommend surveilling for SARS-CoV-2 infections and virus variants, vaccinating at-risk populations, and developing new vaccines. 

## Figures and Tables

**Table 1:** Distribution and comparison of SARS-CoV-2 cases and non-cases in the Borriana COVID-19 cohort; crude relative risk (RR) and 95% confidence interval (CI).

Variables		Cases N = 226	Non-Cases N = 75	Total	RR	95% CI	*p*-Value
Age (years) ± SD ^1^		37.8 ± 17.3	44.8 ± 17.6		0.99	(0.98–0.99)	0.004
Age ≥ 50 years	Yes	57 (22.2)	41 (54.7)	98	0.70	(0.59–0.84)	0.000
	No	169 (74.8)	34 (45.3)	204	1.00		
Male		81 (35.7)	37(49.3)	118	0.87	(0.75–0.99)	0.049
Female		145 (64.3)	38 (50.7)	183	1.00		
Social class ^2^ I–II		50 (22.1)	19(25.3)	69	0.96	(0.81–1.12)	0.581
Social class III–VI		176 (77.9)	56 (74.7)	232	1.00		
Chronic disease	Yes	74(32.7)	30 (40.0)	104	0.92	(0.80–1.07)	0.271
	No	152 (67.3)	45 (60.0)	197	1.00		
Obesity ^3,4^	Yes	30 (13.6)	11 (14.9)	41	0.97	(0.80–1.19)	0.798
	No	190 (86.4)	63 (85.1)	253			
Current smoking ^5^		60 (27.1)	31(41.9)	91	0.76	(0.60–0.97)	0.024
Ex-smoking		38 (17.2)	13 (17.6)	51	0.93	(0.74–1.18)	0.565
Never smoking		123 (55.0)	30 (40.5)	153	1.00		
Alcohol ^6^ intake	Yes	40 (18.0)	15 (21.7)	55	0.96	(0.81–1.15)	0.677
	No	182 (82.0)	59 (78.3)	241	1.00		
Physical exercise ^7^	Yes	135 (60.0)	34 (47.2)	169	1.13	(0.99–1.30)	0.076
	No	90 (40.0)	38 (52.8)	128	1.00		
Numbers of cohabitants at home ^8^ ± SD ^1^		3.3 ± 1.0	2.9 ± 1.1		1.08	(1.02–1.16)	0.011
Family member COVID-19 case ^9^	Yes	196 (89.5)	57 (40.5)	253	1.25	(0.96–1.62)	0.098
	No	23 (10.5)	14 (59.5)	37	1.00		
Exposure to other people at work ^10^	Yes	167 (77.7)	45 (64.3)	212	1.20	(1.00–1.43)	0.049
	No	48 (22.3)	25 (35.7)	73	1.00		
Visiting restaurants/bars ^11^	Yes	158 (72.5)	42 (60.9)	200	1.15	(0.98–1.34)	0.093
	No	60 (27.5)	27 (39.1)	87	1.00		
Face mask wearing ^12^	Yes	69 (32.1)	28 (40.6)	97	0.91	(0.79–1.06)	0.218
	No	146 (67.9)	41 (59.4)	187	1.00		

^1^ SD = standard deviation. ^2^ Social class I–II (upper and middle class), social class III–VI (lower class). ^3^ Obesity = body mass index ≥ 30. ^4^ Missing information, 7 participants. ^5^ Missing information, 6 participants. ^6^ Missing information, 5 participants. ^7^ Missing information, 4 participants. ^8^ Missing information, 6 participants. ^9^ Missing information, 11 participants. ^10^ Missing information, 16 participants. ^11^ Missing information, 14 participants. ^12^ Missing information, 17 participants.

**Table 2:** Distributions and comparison of SARS-CoV-2 vaccines between SARS-CoV-2 cases and non-cases. Robust Poisson regression was conducted, with crude relative risk (RR) and 95% confidence interval (CI).

Variables	Cases N = 226	Non-Cases N = 75	Total	RR	95% CI	*p*-Value
	N (%)	N (%)	N			
Vaccinated with at least 1 dose						
Yes	210 (92.9)	75 (100)	285	0.74	(0.69–0.79)	0.000
No	16 (7.1)	0 (0)	16	1.00		
Completed vaccination						
Vaccinated with 2–3 doses	200 (88.5)	75 (100)	275	0.72	(0.68–0.78)	0.000
Vaccinated with 0–1 dose	26 (11.5)	0 (0)	26	1.00		
Booster vaccination						
Vaccinated with 3 doses	102 (45.1)	63 (84.0)	165	0.68	(0.59–0.77)	0.000
Vaccinated with 0–2 doses	124 (55.9)	12 (16.0)	136	1.00		
Number of vaccines doses received						
0–1 dose	26 (11.5)	0 (0)	26	1.00		
2 doses	98 (43.4)	12 (16.0)	110	0.89	(0.83–0.95)	0.001
3 doses	102 (45.1)	63 (84.0)	165	0.62	(0.55–0.70)	0.000
Type of vaccine						
mRNA ^1^ alone	172 (82.0)	56 (74.7)	228	1.13	(0.93–1.38)	0.222
mRNA ^1^ and other vaccines	38 (18.0)	19 (25.3)	57	1.00		

^1^ SRARS-CoV-2 messenger RNA vaccine.

**Table 3:** Adjusted relative risk (RR) of SARS-CoV-2 and SARS-CoV-2 vaccine effectiveness (VE). Robust Poisson regression was conducted, with 95% confidence interval (CI).

Variables	Adjusted RR 95% CI	Effectiveness 95% CI	*p*-Value
Vaccinated with a least 1 dose	0.78 (0.63–0.96)	22% (4–37)	0.020
Non-vaccinated	1.00		
Completed vaccinated			
Vaccinated with 2–3 doses	0.82 (0.70–0.95)	18% (5–30)	0.011
Vaccinated with 0–1 dose	1.00		
Booster vaccination			
Vaccinated with 3 doses	0.71 (0.61–0.82)	29% (18–39)	0.000
Vaccinated with 0–1–2 doses	1.00		
Number of vaccines doses received			
3 doses	0.63 (0.51–0.78)	37% (22–49)	0.000
2 doses	0.89 (0.76–1.03)	11% (−3–24)	0.149
0–1 dose	1.00		

Adjusted for age, sex, chronic disease, obesity, smoking, alcohol consumption, physical exercise, social class, cohabitants, exposure to other people at work, face mask wearing, visiting restaurants/bars, and family member with COVID-19.

**Table 4:** Crude and adjusted relative risk (RR), and SARS-CoV-2 vaccine effectiveness (VE), stratified by sex, chronic disease, and age. Comparison of 3 doses versus 0–1–2 doses. Robust Poisson regression was conducted, with 95% confidence interval (CI).

Variables	Crude RR 95% CI	Adjusted RR 95% CI	Effectiveness 95% CI	*p*-Value
Female ^1^	0.72 (0.62–0.84)	0.74 (0.63–0.87)	26% (13–37)	0.000
Male ^1^	0.60 (0.48–0.78)	0.62 (0.46–0.83)	38% (17–54)	0.001
Chronic disease				
Yes ^2^	0.70 (0.56–0.87)	0.60 (0.44–0.81)	40% (19–56)	0.006
No ^2^	0.67 (0.56–0.79)	0.74 (0.62–0.89)	26% (11–38)	0.001
Age 50 years and over ^3^	0.60 (0.45–0.80)	0.51 (0.36–0.75)	49% (25–64)	0.000
Age under 50 years ^3^	0.77 (0.67–0.89)	0.77 (0.67–0.88)	23% (12–33)	0.000

^1^ Adjusted for age, chronic disease, obesity, smoking, alcohol consumption, physical exercise, social class, cohabitants, exposure to other people at work, face mask wearing, visiting restaurants/bars, and family member with COVID-19. ^2^ Adjusted for age, sex, obesity, smoking, alcohol consumption, physical exercise, social class, cohabitants, exposure to other people at work, face mask wearing, visiting restaurants/bars, and family member with COVID-19. ^3^ Adjusted for sex, chronic disease, obesity, smoking, alcohol consumption, physical exercise, social class, cohabitants, exposure to other people at work, face mask wearing, visiting restaurants/bars, and family member with COVID-19.

**Table 5:** Comparison of characteristics of symptomatic SARS-CoV-2 cases and non-cases in the Borriana COVID-19 cohort. Relative risk (RR) and 95% confidence interval (CI).

Variables		Cases N = 153	Non-Cases N = 75	Total	RR	95 CI	*p*-Value
		N (%)	N (%)	N			
Age (years) ± SD ^1^		37.1 ± 16.8	44.8 ± 17.6		0.99	(0.98–0.99)	0.002
Age ≥ 50 years	Yes	36 (23.5)	41 (54.7)	77	0.60	(0.47–0.78)	0.000
	No	117 (76.5)	34 (45.3)	151	1.00		
Male		51 (33.3)	37 (49.3)	88	0.80	(0.65–0.98)	0.029
Female		102 (66.7)	38 (50.7)	140	1.00		
Social class ^2^ I–II		36 (23.5)	19(25.3)	55	0.97	(0.78–1.20)	0.769
Social class III–VI		117 (76.5)	56 (74.7)	173	1.00		
Chronic disease	Yes	50 (32.7)	30 (40.0)	80	0.90	(0.73–1.10)	0.294
	No	103 (67.3)	45 (60.0)	148	1.00		
Obesity ^3,4^	Yes	20 (13.5)	11 (14.9)	31	0.96	(0.73–1.27)	0.790
	No	128 (86.5)	63 (85.1)	191			
Current smoking ^5^		39 (26.4)	31(41.9)	70	0.76	(0.60–0.97)	0.024
Ex-smoking		27 (18.2)	13 (17.6)	40	0.92	(0.72–1.18)	0.512
Never smoking		82 (55.4)	30 (40.5)	112	1.00		
Alcohol intake ^6^	Yes	27 (18.1)	15 (21.7)	42	0.95	(0.74–1.22)	0.708
	No	122 (81.9)	59 (78.3)	181	1.00		
Physical exercise ^7^	Yes	88 (57.9)	34 (47.2)	122	1.15	(0.95–1.38)	0.142
	No	64 (42.1)	38 (52.8)	102	1.00		
Numbers of cohabitants at home ^8^ ± SD ^1^		3.3 ± 1.0	2.9 ± 1.1		1.14	(1.04–1.25)	0.007
Family member COVID-19 case ^9^	Yes	137 (90.7)	57 (40.5)	194	1.41	(0.96–2.07)	0.077
	No	14 (9.3)	14 (59.5)	28	1.00		
Exposure to other people at work ^10^	Yes	117 (79.1)	45 (64.3)	162	1.30	(1.01–1.68)	0.041
	No	31 (20.9)	25 (35.7)	56	1.00		
Visiting restaurants/bars ^11^	Yes	111 (74.5)	42 (60.9)	153	1.24	(0.99–1.56)	0.063
	No	38 (25.5)	27 (39.1)	65	1.00		
Face mask wearing ^12^	Yes	42 (28.6)	28 (40.6)	70	0.83	(0.67–1.04)	0.102
	No	105 (71.4)	41 (59.4)	146	1.00		

^1^ SD = standard deviation. ^2^ Social class I–II (upper and middle class), social III–VI (lower class). ^3^ Obesity = body mass index ≥ 30. ^4^ Missing information, 6 participants. ^5^ Missing information, 6 participants. ^6^ Missing information, 5 participants. ^7^ Missing information, 4 participants. ^8^ Missing information, 6 participants. ^9^ Missing information, 6 participants. ^10^ Missing information, 10 participants. ^11^ Missing information, 10 participants. ^12^ Missing information, 12 participants.

**Table 6:** Comparison of SARS-CoV-2 vaccines between symptomatic SARS-CoV-2 cases and non-cases. Robust Poisson regression was conducted, with crude relative risk (RR) and 95% confidence interval (CI).

Variables	Cases N = 153	Non-Cases N = 75	Total	RR	95% CI	*p*-Value
	N (%)	N (%)	N			
Vaccinated with at least 1 dose						
Yes	144 (94.1)	75 (100)	119	0.66	(0.60–0.72)	0.011
No	9 (5.9)	0 (0)	9	1.00		
Completed vaccination						
Vaccinated with 2–3 doses	135 (88.2)	75 (100)	210	0.64	(0.58–0.71)	0.000
Vaccinated with 0–1 dose	18 (11.8)	0 (0)	18	1.00		
Booster vaccination						
Vaccinated with 3 doses	54 (35.3)	63 (84.0)	117	0.51	(0.42–0.64)	0.000
Vaccinated with 0–1–2 doses	99 (64.7)	12 (16.0)	111	1.00		
Number of vaccines doses received						
0–1 dose	18 (11.8)	0 (0)	18	1.00		
2 doses	81 (52.9)	12 (16.0)	93	0.87	(0.81–0.94)	0.001
3 doses	54 (35.3)	63 (84.0)	117	0.46	(0.38–0.56)	0.000
Type of vaccine						
mRNA ^1^ alone	119 (82.8)	56 (74.7)	175	1.20	(0.91–1.58)	0.205
mRNA ^1^ and other vaccines	25 (20.8)	19 (25.3)	44	1.00		

^1^ SRARS-CoV-2 messenger RNA vaccine.

**Table 7:** Adjusted relative risk (RR) and SARS-CoV-2 vaccine effectiveness (VE), comparing vaccinations and doses. Robust Poisson regression was conducted, with 95% confidence interval (CI).

Variable	Adjusted RR 95% CI	Effectiveness 95% CI	*p*-Value
Vaccinated with at least 1 dose	0.81 (0.53–1.22)	19% (−22–47)	0.308
Non-vaccinated	1.00		
Completed vaccinated			
Vaccinated with 2–3 doses	0.82 (0.67–1.01)	18% (−1–33)	0.066
Vaccinated with 0–1 dose	1.00		
Booster vaccination			
Vaccinated with 3 doses	0.54 (0.44–0.68)	46% (32–56)	0.000
Vaccinated with 0–1–2 doses	1.00		
Number of vaccines doses received			
3 doses	0.50 (0.37–0.67)	50% (33–63)	0.000
2 doses	0.92 (0.75–1.12)	8% (−12–25)	0.376
0–1 dose	1.00		

Adjusted for age, sex, chronic disease, obesity, smoking, alcohol consumption, physical exercise, social class, cohabitants, exposure to other people at work, face mask wearing, visiting restaurants/bars, and family member with COVID-19.

**Table 8:** Crude (RR) and adjusted relative risk (aRR) and SARS-CoV-2 vaccine effectiveness (VE), stratified by sex, chronic disease, and age. Comparisons between vaccinations with 3 doses versus 0–1–2 doses. Robust Poisson regression was conducted, with 95% confidence interval (CI).

Variable	Crude RR 95% CI	Adjusted RR 95% CI	Effectiveness 95% CI	*p*-Value
Female ^1^	0.59 (0.46–0.74)	0.61 (0.49–0.78)	39% (22–51)	0.000
Male ^1^	0.42 (0.28–0.63)	0.38 (0.23–0.63)	62% (37–77)	0.000
Chronic disease				
Yes ^2^	0.60 (0.44–0.81)	0.48 (0.32–0.73)	52% (27–68)	0.001
No ^2^	0.47 (0.35–0.63)	0.55 (0.41–0.73)	45% (27–59)	0.001
Age 50 years and over ^3^	0.46 (0.31–0.69)	0.40 (0.24–0.64)	60% (36–76)	0.000
Age under 50 years ^3^	0.61 (0.48–0.78)	0.61 (0.48–0.77)	39% (23–52)	0.000

^1^ Adjusted for age, chronic disease, obesity, smoking, alcohol consumption, physical exercise, social class, cohabitants, exposure to other people at work, face mask wearing, visiting restaurants/bars, and family member with COVID-19. ^2^ Adjusted for age, sex, obesity, smoking, alcohol consumption, physical exercise, social class, cohabitants, exposure to other people at work, face mask wearing, visiting restaurants/bars, and family member with COVID-19. ^3^ Adjusted for sex, chronic disease, obesity, smoking, alcohol consumption, physical exercise, social class, cohabitants, exposure to other people at work, face mask wearing, visiting restaurants/bars, and family member with COVID-19.

**Table 9:** Comparison between first approach (all SARS-CoV-2 cases) and second approach: adjusted relative risk (aRR), stratification, vaccine effectiveness (VE), and 95% confidence interval (CI).

Measures	First Approach	Second Approach
	All SARS-CoV-2 Cases	Symptomatic SARS-CoV-2 Cases
Variables	aRR (95% CI)	aRR (95% CI)
Vaccinated with at least 1 dose	0.78 (0.63–0.96)	0.81 (0.53–1.22)
Vaccinated with 2–3 doses	0.82 (0.70–0.95)	0.82 (0.67–1.01)
Vaccinated with 3 doses	0.71 (0.61–0.82)	0.54 (0.44–0.68)
Number of vaccine doses received		
3 doses	0.63 (0.51–0.78)	0.50 (0.37–0.67)
2 doses	0.89 (0.76–1.03)	0.92 (0.75–1.12)
Effectiveness	VE (95% CI)	VE (95% CI)
Vaccinated with at least one dose	22% (4–37)	19% (−22–47)
Vaccinated with 2–3 doses	18% (5–30)	18% (−1–33)
Vaccinated with 3 doses	29% (18–39)	46% (32–56)
Number of vaccine doses received		
3 doses	37% (22–49)	50% (33–63)
2 doses	11% (−3–24)	8% (−12–25)
Stratification	aRR (95% CI)	aRR (95% CI)
Female	0.74 (0.63–0.87)	0.62 (0.49–0.78)
Male	0.62 (0.46–0.83)	0.38 (0.23–0.63)
Chronic disease		
Yes	0.60 (0.44–0.81)	0.48 (0.32–0.72)
No	0.74 (0.62–0.89)	0.55 (0.42–0.74)
Age 50 years and above	0.51 (0.36–0.75)	0.41 (0.26–0.66)
Age under 50 years	0.77 (0.67–0.88)	0.61 (0.47–0.77)
Effectiveness	VE (95% CI)	VE (95% CI)
Female	26% (13–37)	39% (22–51)
Male	38% (13–54)	62% (37–77)
Chronic disease		
Yes	40% (19–56)	52% (27–68)
No	26% (11–38)	45% (27–59)
Age 50 years and above	49% (25–64)	60% (36–76)
Age under 50 years	23% (12–33)	39% (23–53)

## Data Availability

The data presented in this study are available on request from the corresponding author due to official authorizations.

## Supplementary Materials

The following supporting information can be downloaded at: https://www.mdpi.com/article/10.3390/epidemiologia7010001/s1, Supplement S1. Robust Poisson regression models.
