# Supplementary material for: SARS-CoV-2 mRNA Vaccine Effectiveness in the Borriana COVID-19 Cohort: A Prospective Population-Based Cohort Study
Source: Epidemiologia (Basel). 2025 Dec 19;7(1):1. doi: 10.3390/epidemiologia7010001 (PMC12821481; doi:10.3390/epidemiologia7010001)
Supplement: Supplementary file 1 [file epidemiologia-07-00001-s001.zip › epidemiologia-3995693-supplementary.pdf]

Supplement S1:

Title: Robust Poisson regression models

Stata ® 14 version 2

Commands:
poisson
irr = relative risk
r=robust
stat gof: statistic good-of-fit tests

Variables:

Endcase2022: SARS-CoV-2 infection
vaccinated: Yes =1 No=0
vaccinated2: 2-3 doses vaccine: Yes =1 No=0
dummy variables number of vaccine doses
dd4: vaccinated 3 doses vaccine: Yes=1 No=0
dd3: vaccinated 2 doses vaccine .Yes=1 No=0
dd2: vaccinated 1 doses vaccine. Yes=1 No=0
dd1: vaccinated 0 doses vaccine. Yes=1 No=0
sex: Male=1 Female=0
age2022: number of years
disease: chronic disease Yes=1 No=0c
class: social class I-II=1 social class III-VI=0
oo4final: obesity Yes=1 No=0
fumfinal: smoking: Never=0 Ex smoking =1 Somking=2
ohfinal alcohol consumption Yes=1 No=0
convivientes: number of cohabitants at home
acudeterrabar: visiting restauarants/bar Yes=1 No=0
masca: face mask wearing Yes=1 No=0
fit2022: physical exercise Yes=1 No=0
contact: exposure to public at work Yes=1 No=0
convv: family COVID-19 case Yes=1 No=0
vacunaantescase= vaccinated before SARS-CoV-2 infection
newasintomatic = asymptomatic SARS-CoV-2 infection=0 (asymptomatic excluded)

```
poisson endcase2022 vaccinated sex age2022 disease  class oo4final fumfinal ohfinal convivientes
acudeterrares masca fit2022 contact convv if vacunaantescase==1, irr r

Iteration 0:   log pseudolikelihood = -240.82836
Iteration 1:   log pseudolikelihood = -240.82835

Poisson regression                    Number of obs     =        253
```

```
                                      Wald chi2(14)    =      36.37
                                      Prob > chi2      =     0.0009
Log pseudolikelihood = -240.82835             Pseudo R2        =     0.0169

------------------------------------------------------------------------------
             |               Robust
 endcase2022 |        IRR   Std. Err.      z    P>|z|     [95% Conf. Interval]
-------------+----------------------------------------------------------------
  vaccinated |     .77554   .0849938    -2.32   0.020     .6256312    .9613688
         sex |   .8006492   .0650219    -2.74   0.006     .6828337    .9387925
     age2022 |   .9968696   .0027975    -1.12   0.264     .9914017    1.002368
     disease |   1.064573   .0901724     0.74   0.460     .9017297    1.256825
       class |   1.034011   .0812813     0.43   0.670     .8863682    1.206247
    oo4final |   1.091789   .1278142     0.75   0.453     .8679397     1.37337
     fumfinal |   .9419064   .0415524    -1.36   0.175     .8638868    1.026972
     ohfinal |   1.117511   .0949327     1.31   0.191     .9461114    1.319963
 convivientes |   1.066724   .0416429     1.65   0.098     .9881498    1.151546
acudeterraresto |   1.157453   .0998386     1.70   0.090     .9774198    1.370647
       masca |   .9599398   .0770218    -0.51   0.610     .8202513    1.123417
     fit2022 |   1.203392   .0904345     2.46   0.014      1.03858    1.394359
     contact |   1.078367   .1117329     0.73   0.467     .8801791    1.321181
       convv |   1.183678   .1578553     1.26   0.206     .9114175    1.537269
       _cons |   .6371442   .1367786    -2.10   0.036     .4183179    .9704407
------------------------------------------------------------------------------

. estat gof

         Deviance goodness-of-fit =  97.65671
         Prob > chi2(238)         =    1.0000

         Pearson goodness-of-fit  =  60.53494
         Prob > chi2(238)         =    1.0000

.   poisson endcase2022 vaccinated2 sex age2022 disease  class oo4final fumfinal ohfinal convivientes
acudeterrares masca fit202 contact convv if vacunaantescase==1, irr r

Iteration 0:   log pseudolikelihood = -240.80006
Iteration 1:   log pseudolikelihood = -240.80005

Poisson regression                              Number of obs    =        253
                                      Wald chi2(14)    =      40.36
                                      Prob > chi2      =     0.0002
Log pseudolikelihood = -240.80005             Pseudo R2        =     0.0170

------------------------------------------------------------------------------
             |               Robust
 endcase2022 |        IRR   Std. Err.      z    P>|z|     [95% Conf. Interval]
-------------+----------------------------------------------------------------
 vaccinated2 |   .8157738   .0649143    -2.56   0.011     .6979693    .9534615
```

```
             sex |   .8009929   .0647456    -2.75   0.006     .6836356    .9384966
         age2022 |   .9969709   .0027877    -1.08   0.278      .991522     1.00245
         disease |   1.070394   .0910269     0.80   0.424     .9060598    1.264533
           class |   1.029083   .0805805     0.37   0.714     .8826706    1.199782
        oo4final |   1.092682   .1277757     0.76   0.448     .8688726    1.374141
        fumfinal |   .9442111   .0417216    -1.30   0.194     .8658791    1.029629
         ohfinal |   1.116615   .0946151     1.30   0.193     .9457537    1.318345
    convivientes |   1.066769   .0417059     1.65   0.098     .9880801    1.151724
acudeterraresto |   1.153486   .0991449     1.66   0.097     .9746521    1.365134
           masca |    .959034   .0769841    -0.52   0.602     .8194187    1.122437
         fit2022 |   1.203884   .0905051     2.47   0.014     1.038947    1.395006
         contact |   1.073054   .1097825     0.69   0.491     .8780851    1.311314
           convv |   1.175139   .1558133     1.22   0.224     .9062066    1.523882
           _cons |   .6067418   .1356965    -2.23   0.025     .3914123    .9405315
------------------------------------------------------------------------------

. estat gof

         Deviance goodness-of-fit =  97.60011
         Prob > chi2(238)         =    1.0000

         Pearson goodness-of-fit  =  60.49875
         Prob > chi2(238)         =    1.0000

.   poisson endcase2022 dd4 sex age2022 disease  class oo4final fumfinal ohfinal convivientes
acudeterrares masca fit2022 contact convv if vacunaantescase==1, irr r

Iteration 0:   log pseudolikelihood = -238.81744
Iteration 1:   log pseudolikelihood = -238.81744

Poisson regression                              Number of obs     =        253
                                                Wald chi2(14)     =      51.40
                                                Prob > chi2       =     0.0000
Log pseudolikelihood = -238.81744               Pseudo R2         =     0.0251

------------------------------------------------------------------------------
             |               Robust
 endcase2022 |        IRR   Std. Err.      z    P>|z|     [95% Conf. Interval]
-------------+----------------------------------------------------------------
             dd4 |   .7075162   .0513624    -4.77   0.000     .6136815    .8156986
             sex |    .810768   .0638999    -2.66   0.008     .6947203    .9462006
         age2022 |   1.000889    .002758     0.32   0.747     .9954976    1.006309
         disease |   1.054112    .089173     0.62   0.533     .8930563    1.244212
           class |   1.023521   .0789925     0.30   0.763     .8798392    1.190666
        oo4final |   1.105258   .1230416     0.90   0.369      .888596    1.374746
        fumfinal |   .9362172   .0404863    -1.52   0.127     .8601354    1.019029
         ohfinal |   1.102458   .0912993     1.18   0.239      .937282    1.296742
    convivientes |   1.059712   .0406949     1.51   0.131     .9828797    1.142551
acudeterraresto |   1.159256   .0971196     1.76   0.078     .9837118    1.366127
```

masca	.9944532	.078336	-0.07	0.944	.8521827	1.160476
fit2022	1.188616	.0851203	2.41	0.016	1.032963	1.367725
contact	1.092292	.1058716	0.91	0.362	.9033069	1.320816
convv	1.186155	.146464	1.38	0.167	.9311863	1.510936
_cons	.51505	.1178535	-2.90	0.004	.3289106	.8065307

. estat gof

Deviance goodness-of-fit = 93.63488
Prob > chi2(238) = 1.0000

Pearson goodness-of-fit = 59.70838
Prob > chi2(238) = 1.0000

. poisson endcase2022 dd4 dd3 sex age2022 disease class oo4final fumfinal ohfinal convivientes acudeterrares masca fit2022 contact convv if vacunaantescase==1, irr r

Iteration 0: log pseudolikelihood = -238.74547
Iteration 1: log pseudolikelihood = -238.74547

Poisson regression Number of obs = 253
Wald chi2(15) = 53.92
Prob > chi2 = 0.0000
Log pseudolikelihood = -238.74547 Pseudo R2 = 0.0254

endcase2022	IRR	Robust Std. Err.	z	P>|z|	[95% Conf.	Interval]
dd4	.6315056	.0672291	-4.32	0.000	.5125775	.7780273
dd3	.8863541	.0676238	-1.58	0.114	.7632475	1.029317
sex	.8074214	.063794	-2.71	0.007	.6915876	.942656
age2022	1.001375	.0028744	0.48	0.632	.9957572	1.007025
disease	1.057772	.0896461	0.66	0.508	.895886	1.248911
class	1.024295	.0792754	0.31	0.756	.880129	1.192076
oo4final	1.107088	.1233683	0.91	0.361	.8898737	1.377322
fumfinal	.9386796	.0407883	-1.46	0.145	.8620457	1.022126
ohfinal	1.100992	.0910298	1.16	0.245	.9362829	1.294677
convivientes	1.058188	.0408999	1.46	0.143	.9809872	1.141465
acudeterraresto	1.155319	.0961308	1.74	0.083	.9814676	1.359966
masca	.9936914	.0782889	-0.08	0.936	.8515081	1.159616
fit2022	1.195317	.0864359	2.47	0.014	1.037364	1.377322
contact	1.100614	.1068897	0.99	0.324	.9098455	1.33138
convv	1.189443	.1460093	1.41	0.158	.9350931	1.512977
_cons	.5602817	.1231786	-2.64	0.008	.3641404	.8620731

. estat gof

Deviance goodness-of-fit = 93.49093
Prob > chi2(237) = 1.0000

Pearson goodness-of-fit = 59.6915
Prob > chi2(237) = 1.0000

. poisson endcase2022 vaccinated sex age2022 disease class oo4final fumfinal ohfinal convivientes acudeterrares masca fit2022 contact convv if vacunaantescase==1&newasintomatic==0, irr r

Iteration 0: log pseudolikelihood = -175.70139
Iteration 1: log pseudolikelihood = -175.70135
Iteration 2: log pseudolikelihood = -175.70135

Poisson regression Number of obs = 192
Wald chi2(14) = 35.01
Prob > chi2 = 0.0015
Log pseudolikelihood = -175.70135 Pseudo R2 = 0.0297

endcase2022	IRR	Robust Std. Err.	z	P>|z|	[95% Conf.	Interval]
vaccinated	.8060506	.1705964	-1.02	0.308	.5323656	1.220435
sex	.7258045	.0821624	-2.83	0.005	.5813828	.9061022
age2022	.9950596	.0039237	-1.26	0.209	.9873989	1.00278
disease	1.093192	.1243664	0.78	0.434	.8747019	1.366259
class	1.03215	.1113479	0.29	0.769	.8354404	1.275175
oo4final	1.110814	.1828263	0.64	0.523	.8045337	1.533694
fumfinal	.9037008	.0573457	-1.60	0.111	.7980138	1.023385
ohfinal	1.212823	.1426359	1.64	0.101	.9631422	1.527229
convivientes	1.093914	.0590839	1.66	0.097	.9840306	1.216068
acudeterraresto	1.178313	.146365	1.32	0.187	.9236934	1.503118
masca	.9280333	.1062059	-0.65	0.514	.7415671	1.161386
fit2022	1.214706	.1205449	1.96	0.050	.9999995	1.475512
contact	1.070984	.1511486	0.49	0.627	.8121799	1.412256
convv	1.376147	.2797467	1.57	0.116	.9239119	2.049743
_cons	.4958223	.1768594	-1.97	0.049	.2464355	.9975828

. estat gof

Deviance goodness-of-fit = 89.4027
Prob > chi2(177) = 1.0000

Pearson goodness-of-fit = 60.18882
Prob > chi2(177) = 1.0000

. poisson endcase2022 vaccinated2 sex age2022 disease class oo4final fumfinal ohfinal convivientes

```
acudeterrares masca fit2022 contact convv if vacunaantescase==1&newasintomatic==0, irr r

Iteration 0:   log pseudolikelihood = -175.62639
Iteration 1:   log pseudolikelihood = -175.62635
Iteration 2:   log pseudolikelihood = -175.62635

Poisson regression                              Number of obs     =        192
                                                Wald chi2(14)     =      40.36
                                                Prob > chi2       =     0.0002
Log pseudolikelihood = -175.62635               Pseudo R2         =     0.0301

------------------------------------------------------------------------------
             |               Robust
  endcase2022 |        IRR   Std. Err.      z    P>|z|     [95% Conf. Interval]
-------------+----------------------------------------------------------------
  vaccinated2 |   .8225708   .0874206    -1.84   0.066     .6678975    1.013064
          sex |   .7242019   .0814133    -2.87   0.004      .580991    .9027136
      age2022 |   .9954088   .0039145    -1.17   0.242      .987766    1.003111
      disease |   1.102304   .1239372     0.87   0.386     .8842943    1.374061
        class |   1.026492   .1104744     0.24   0.808     .8312781    1.267548
     oo4final |     1.1108   .1827342     0.64   0.523      .804651    1.533432
     fumfinal |   .9069842   .0578246    -1.53   0.126      .800445    1.027704
      ohfinal |   1.209383    .141277     1.63   0.104     .9618971    1.520544
 convivientes |   1.092941   .0593192     1.64   0.102      .982648    1.215614
acudeterraresto |   1.173205   .1440266     1.30   0.193     .9223109    1.492348
        masca |   .9269353   .1056033    -0.67   0.505     .7414371    1.158843
      fit2022 |   1.217949   .1206294     1.99   0.047     1.003052    1.478886
      contact |   1.069827   .1504258     0.48   0.631     .8121352    1.409286
        convv |   1.365568   .2776471     1.53   0.125     .9167419    2.034134
        _cons |   .4802041   .1625412    -2.17   0.030     .2473478     .932274
------------------------------------------------------------------------------

. estat gof

         Deviance goodness-of-fit =  89.25271
         Prob > chi2(177)         =    1.0000

         Pearson goodness-of-fit  =  60.15528
         Prob > chi2(177)         =    1.0000

.    poisson endcase2022 dd4 sex age2022 disease  class oo4final fumfinal ohfinal convivientes
acudeterrares masca fit2022 contact convv if vacunaantescase==1&newasintomatic==0, irr r

Iteration 0:   log pseudolikelihood = -171.18493
Iteration 1:   log pseudolikelihood =  -171.1849
Iteration 2:   log pseudolikelihood =  -171.1849

Poisson regression                              Number of obs     =        192
                                                Wald chi2(14)     =      69.97
```

```
                                   Prob > chi2       =     0.0000
Log pseudolikelihood =  -171.1849              Pseudo R2         =     0.0547

------------------------------------------------------------------------------
             |               Robust
   endcase2022 |      IRR   Std. Err.      z    P>|z|     [95% Conf. Interval]
----------------+----------------------------------------------------------------
          dd4 |   .5423839   .0610241    -5.44   0.000     .4350481    .6762018
          sex |   .7368512   .0775567    -2.90   0.004     .5994973    .9056751
      age2022 |     1.0029    .003824     0.76   0.448     .9954332    1.010423
      disease |   1.108628   .1261926     0.91   0.365     .8869423    1.385722
        class |   1.001727   .1027052     0.02   0.987     .8193646    1.224677
     oo4final |   1.127667   .1716109     0.79   0.430     .8368408    1.519562
     fumfinal |   .9030704   .0553536    -1.66   0.096     .8008429    1.018347
      ohfinal |   1.159554   .1262486     1.36   0.174     .9367311    1.435381
 convivientes |   1.092411   .0576529     1.67   0.094     .9850613     1.21146
acudeterraresto |   1.151611   .1347501     1.21   0.228     .9156017    1.448455
        masca |   .9882512   .1078443    -0.11   0.914     .7979558    1.223928
      fit2022 |   1.179378   .1092792     1.78   0.075     .9835177    1.414243
      contact |   1.110217   .1391178     0.83   0.404     .8684534    1.419284
        convv |   1.389256   .2325724     1.96   0.050     1.000655    1.928768
        _cons |   .3800276   .1194783    -3.08   0.002     .2052119    .7037651
------------------------------------------------------------------------------

. estat gof

         Deviance goodness-of-fit =   80.3698
         Prob > chi2(177)         =    1.0000

         Pearson goodness-of-fit  =  57.46451
         Prob > chi2(177)         =    1.0000

.    poisson endcase2022 dd4 dd3 sex age2022 disease  class oo4final fumfinal ohfinal convivientes
acudeterrares masca fit2022 contact convv if vacunaantescase==1&newasintomatic==0, irr r

Iteration 0:   log pseudolikelihood = -171.15754
Iteration 1:   log pseudolikelihood =  -171.1575
Iteration 2:   log pseudolikelihood =  -171.1575

Poisson regression                              Number of obs     =        192
                                   Wald chi2(15)     =      71.36
                                   Prob > chi2       =     0.0000
Log pseudolikelihood =  -171.1575              Pseudo R2         =     0.0548

------------------------------------------------------------------------------
             |               Robust
   endcase2022 |      IRR   Std. Err.      z    P>|z|     [95% Conf. Interval]
----------------+----------------------------------------------------------------
          dd4 |   .4987094   .0768788    -4.51   0.000     .3686636    .6746289
```

```
             dd3 |   .9162009   .0922895    -0.87   0.385     .7520534    1.116176
             sex |    .736933   .0771853    -2.91   0.004     .6001701    .9048604
         age2022 |   1.003232   .0039567     0.82   0.413     .9955072    1.011017
         disease |   1.109909   .1262868     0.92   0.359     .8880488    1.387197
           class |   1.000993   .1028007     0.01   0.992     .8184907    1.224189
        oo4final |   1.128772   .1716972     0.80   0.426     .8377801    1.520835
        fumfinal |   .9051584   .0557823    -1.62   0.106      .802172    1.021367
         ohfinal |   1.157441   .1258536     1.34   0.179     .9352855    1.432365
    convivientes |    1.09104   .0580348     1.64   0.101     .9830222    1.210927
acudeterraresto |   1.151319    .134198     1.21   0.227     .9161768    1.446811
           masca |   .9884046   .1080463    -0.11   0.915     .7977865    1.224568
         fit2022 |    1.18464   .1108643     1.81   0.070     .9861138    1.423135
         contact |   1.112453   .1391435     0.85   0.394      .870593    1.421505
           convv |   1.386944   .2319323     1.96   0.050     .9993468     1.92487
           _cons |   .4065491   .1279005    -2.86   0.004     .2194445    .7531844
------------------------------------------------------------------------------

. estat gof

         Deviance goodness-of-fit =  80.31501
         Prob > chi2(176)         =    1.0000

         Pearson goodness-of-fit  =  57.45593
         Prob > chi2(176)         =    1.0000

.
```
